# Sensing phases of water via nitrogen-vacancy centres in diamond

**DOI:** 10.1038/s41598-018-31745-3

**Published:** 2018-09-07

**Authors:** P. Fernández-Acebal, M. B. Plenio

**Affiliations:** Institut für Theoretische Physik and Center for Integrated Quantum Science and Technology (IQST), Albert-Einstein Allee 11, Universitat Ulm, 89069 Ulm, Germany

## Abstract

Ultra-thin layers of liquids on a surface behave differently from bulk liquids due to liquid-surface interactions. Some examples are significant changes in diffusion properties and the temperature at which the liquid-solid phase transition takes place. Indeed, molecular dynamics simulations suggest that thin layers of water on a diamond surface may remain solid even well above room temperature. However, because of the small volumes that are involved, it is exceedingly difficult to examine these phenomena experimentally with current technologies. In this context, shallow NV centres promise a highly sensitive tool for the investigation of magnetic signals emanating from liquids and solids that are deposited on the surface of a diamond. Moreover, NV centres are non-invasive sensors with extraordinary performance even at room-temperature. To that end, we present here a theoretical work, complemented with numerical evidence based on bosonization techniques, that predicts the measurable signal from a single NV centre when interacting with large spin baths in different configurations. In fact, by means of continuous dynamical decoupling, the polarization exchange between a single NV centre and the hydrogen nuclear spins from the water molecules is enhanced, leading to differences in the coherent dynamics of the NV centre that are interpreted as an unambiguous trace of the molecular structure. We therefore propose single NV centres as sensors capable to resolve structural water features at the nanoscale and even sensitive to phase transitions.

## Introduction

Water is often referred to as one of the key molecules for life. Indeed, it takes a central role in many biological and chemical systems. Still, several properties of water remain under discussion; especially, the behaviour of water at interfaces^1–3^. In particular, the hydrophilic behaviour of biocompatible substrates has seen a significant increase in attention in recent years, with many studies reporting nucleation and ordering of water molecules on highly hydrophilic surfaces^4–6^. Widely used methods of investigation, such as X-ray diffraction (XRD), neutron scattering or nuclear magnetic resonance (NMR), are limited by their spatial resolution and are highly affected by the thermal broadening. These disadvantages may be overcome using high-resolution techniques such as atomic force microscopy (AFM), scanning tunnelling microscope (STM) or similar^7^. Still, the mentioned methods require low temperatures and high vacuum, making it hard to study the ordering of water molecules at ambient conditions, which is the interesting regime for most applications, especially biology and medicine. In parallel, during the last decades, new diamond production techniques have opened the route to novel applications in medicine and biology, making it a valuable candidate to substitute the commonly-used materials of titanium and stainless steel in medical prosthetics^8–10^. Yet, biocompatibility of diamond is poor since it may abrade internal tissues^11^, and a biocompatible covering such as water may confer diamond the possibility to be used in such applications.

In this study we propose a sensing protocol based on shallowly implanted nitrogen-vacancy (NV) centres in bulk diamond that will help to detect the creation of thin water-ice layers covering the diamond surface. The designed technique is non-invasive, thus does not interfere with chemical processes occurring in water, and capable of determining the phase and behaviour of water under ambient conditions. Our sensing tool consists on an NV centre which possesses an electronic spin-1 in its ground state that can be easily polarized and read-out by optical means^12^. The NV centre electronic spin is photostable and exhibits long coherence times at room-temperature even when shallowly implanted in bulk diamond^13–16^. In fact, single nuclear spin sensing of immobilized spins has been demonstrated to be feasible within seconds^17^, and furthermore single nuclear spins can be detected in a similar time-scale even when undergoing random thermal motion^18^. Moreover, NMR measurements with chemical-shift resolutions have recently been achieved^19–21^. Consequently, the NV centre is an ideal candidate for detecting and sensing of dense nuclear baths, as has been shown using decoherence measurement of electronic spins^22,23^.

Here we examine firstly spin decoherence produced by a given bath and secondly the direct population exchange between the NV centre and the surrounding nuclei in the Hartmann-Hahn double resonance (HHDR) regime^24^. Within this regime, the NV centre is first driven with a microwave field, inducing Rabi oscillations on its electronic spin. Polarization transfer occurs when the Rabi frequency is set equal to the energy splitting of certain nuclear spins. In this scheme, the interaction with a given set of nuclei is enhanced while possible interactions with undesired nuclear species, that is, those with different energy splitting, are suppressed^25,26^. The HHDR performance dramatically depends on the nuclear motion, which is determined by the water phase. Therefore, measuring the NV centre population indicates whether water is liquid or solid. In fact, continuous dynamical decoupling based on HHDR has proved its efficiency in the sensing of both single and large spin baths^26,27^. Moreover, HHDR protocols are limited by the relaxation time in the rotating frame *T*_1*ρ*_^28,29^ that is typically in the order of milliseconds^15^, which results in significat advantages for our proposal. In addition, our theoretical analysis is better suitable for HHDR-based protocols than pulse based schemes^30–32^.

This paper is organized as follows. First, our theoretical set-up is described. We outline the required substrate for ice to be stable and introduce the NV centre as a sensor for different water phases. Second, we develop a theory that predicts the measurable signal from an NV centre spin interacting with the three plausible baths either liquid, solid or a mixture of both. Our approach combines a purely analytical prediction for fast diffusing spin with a bosonization protocol for the simulation of stationary nuclei, the latter of which helps us to obtain numerical predictions. Finally, both numerical and theoretical results are presented and compared, suggesting that phase differentiation is possible in a short time-scale at room-temperature even for ultra-thin water layers. Moreover, we show that applying a magnetic field gradient along the quantization axis of the NV centre we can obtain a faithful description of the crystal structure of solid ice.

## System Description

According to^33,34^, water ice may be stable on a (111) diamond surface even above room temperature, since this surface has minimal mismatch with ice Ih crystal lattice. In fact, this lattice similarity reduces the structural strain, thus helping ice to stabilize. This effect is inherent to the (111) surface and is not expected for (100) nor (110). Yet, strain reduction alone does not ensure a proper stabilization^35^ as is clear from the fact that diamond is indeed hydrophobic. In light of the results reported in^34^, the hydrophilic behaviour is increased when the surface is chemically modified using atoms with high water affinity. Hence, we presume here an alkali based surface termination where 1/3 of diamond terminations are synthetically substituted by Na, while the remaining external carbons are saturated by F atoms. Other surface terminations have been numerically proven to give rise to higher stabilization such as Na-H^33^, which is expected to help to stabilize several ice layers. However, in this model hydrogen-based surface terminations are not considered since they interfere with the signal emanating from water protons. Also, even in the absence of H, the surface termination itself may adversely affect the charge state of the NV centre^36–38^. Still, specific surface terminations may be engineered in order to help charge state stabilization^39^, and specially surface termination based on F atoms are expected to favour negatively-charged NV centres^40,41^. Therefore, throughout this work we assume a surface termination based on Na-F^33^ on the diamond surface, yet we do not consider charge-state transitions, and moreover we assume that this Na-F layer does not play any significant role in the presented NV dynamics.

**Figure 1:**
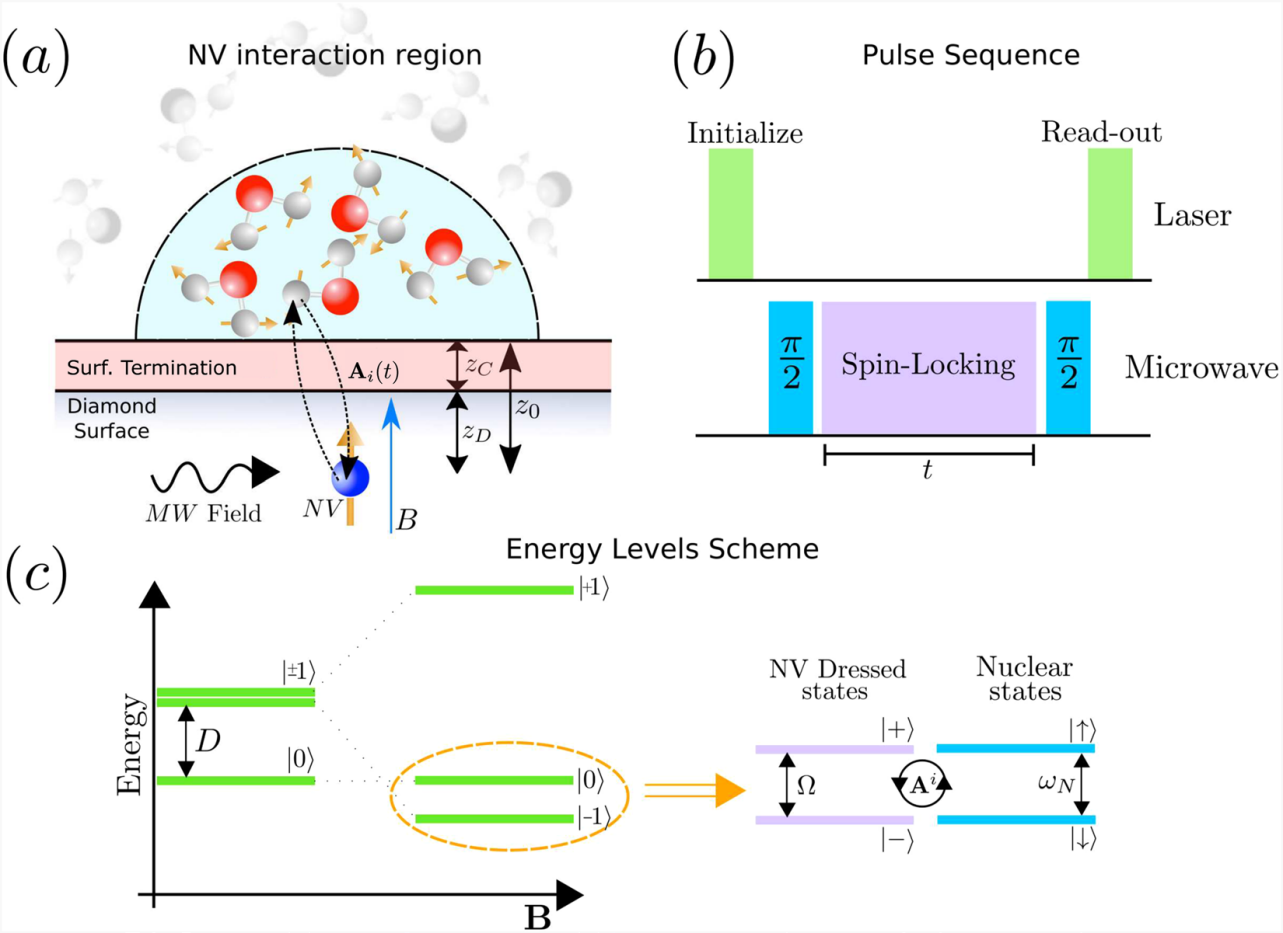
Schematic set-up for water phase sensing with shallow NV centres in bulk diamond and important parameters. (**a**) The NV centre is located at a distant *z*_0_ beneath the diamond surface. The water molecules are deposited on top of the diamond. The ^1^*H* spins conforming the water move stochastically when liquid, stay fix when solid and may stay fixed in few layers while diffusing on top when the two phases coexists. The NV centre interacts via dipole-dipole magnetic interaction, with a strength *g*_*i*_. (**b**) The pulse sequence applied to measure the NV polarization loss. The NV is initially polarized and read-out using green-laser. During the microwave driving, *t*, the NV interacts with the nuclear spins fulfilling the HHDR condition. (**c**) Energy levels of the NV centre and nuclear system. In the absence of magnetic field the NV is a spin–1 systems with projections *m*_*s*_ = 0, ±1, even at zero-field it exist a splitting of *D* = 2.87 GHz. Degeneracy between *m*_*s*_ = ±1 levels is lifted applying an external magnetic field, *B*, parallel to the NV axis. The transition between *m*_*s*_ = 0 and *m*_*s*_ = −1 is driven with microwave field inducing a Rabi frequency Ω, which matches the nuclear Larmor frequency *ω*_*N*_ = *γ*_*N*_*B* permitting polarization transfer.

The sensing protocol is performed using a single shallowly implanted NV centre in bulk diamond, Fig. 1. The NV is assumed to be placed at a distant *z*_0_ ≈ 3 nm beneath the surface. Water is deposited on top of the chemically modified diamond at a temperature that ensures ice formation. Then, the system is heated up to room temperature, which is our regime of interest, and finally the experimental measurements are performed. The polarization loss of the NV centre is straightforwardly measured using a spin-locking sequence as depicted in Fig. 1(b). The pulse-sequence proceeds as follows. First, the NV is optically polarized using 532 nm laser light. Next, a *π*/2 pulse is applied and a suitable microwave field is used in order to provide a spin-locking such that the HHDR condition is fulfilled. During this period the NV exchanges its polarization with a selected nuclear species and its environment. Finally, a second *π*/2 pulse rotates back the NV centre spin and its state is measured by fluorescence spectroscopy.

Now we consider the measurable signal from the NV centre emanating from three plausible scenarios. First, the water becomes liquid at room-temperature, suggesting the chemically modified diamond is hydrophobic and does not serve as ice-substrate. Second, the water remains frozen, indicating the hydrophilic behaviour of the surface. Third and more realistic, only few layers of ice are stabilized on top of the diamond while the remainder becomes liquid.

## System Hamiltonian

Our system comprises a single NV centre interacting with *N* hydrogen spins-1/2 via dipole-dipole magnetic interaction. The NV centre possesses an electronic spin-1 with projections *m*_*s*_ = 0, ±1. Applying an external magnetic field, **B**, parallel to the NV quantization axis the degeneracy between *m*_*s*_ = ±1 levels is lifted. Additionally, using a MW field resonant with the *m*_*s*_ = 0 → *m*_*s*_ = −1 transition allows us to consider the NV as an effective qubit. Furthermore, the external field, **B**, induces a Larmor frequency on the nuclear spins *ω*_*N*_ = *γ*_*N*_|**B**|, where *γ*_*N*_ is the ^1^*H* gyromagnetic ratio. In the dressed state basis for the NV, $|\,\pm \rangle =\frac{1}{\sqrt{2}}(|0\rangle \pm |\,-\,1\rangle )$, the Hamiltonian of the system in the secular approximation with respect to the NV reads (*ħ* = 1)^26,27^

1 $$H={\rm{\Omega }}{S}_{z}+\sum _{i=1}^{N}\,{\omega }_{N}{I}_{z}^{i}+({S}_{x}-\frac{1}{2})\,\sum _{i=1}^{N}\,{{\bf{A}}}^{i}({{\bf{r}}}_{i}){{\bf{I}}}^{i}+{H}_{N-N},$$

where Ω is the NV centre Rabi frequency, **S** is the spin-1/2 operator in the dressed state basis, **I**^*i*^ is the spin operator of the *i*–th nucleus and **A**^*i*^(*t*) is the hyperfine vector between the NV centre and the *i*–th nuclear spin, which depends on the relative position between NV and nuclei, **r**_*i*_, and *H*_*N*,*N*_ is the internuclear coupling. Notice that several processes originate from the dipole-dipole interaction namely, flip-flop along with flip-flip exchange and dephasing on the nuclear spin (see Supplementary Material). Now we consider the three plausible scenarios of liquid water, solid water and of coexisting phases.

### First scenario: Liquid water

We first assume that water remains liquid at room temperature, corresponding to water on hydrophobic substrates. In this scenario, nuclear motion is fast, hence internuclear interactions represented by *H*_*N*–*N*_ average out to become negligible. Besides, nuclear random motion leads to a time-dependent stochastic coupling, **A**^*i*^(**r**_*i*_(*t*)), with certain correlation time *τ*_*c*_, which depends on the water diffusion coefficient and the NV depth as ${\tau }_{c}\propto {z}_{0}^{2}/{{\mathscr{D}}}_{W}$^27,42^.

For water at room temperature the diffusion coefficient is large, ${{\mathscr{D}}}_{W}\approx 2\cdot {10}^{3}\,{{\rm{nm}}}^{2}\,\mu \,{{\rm{s}}}^{-1}$, which in combination with a shallowly implanted NV centre leads to, *τ*_*c*_ ≈ 2 ns. Consequently, for time scales exceeding *τ*_*c*_, the system state is described by $\langle \rho \rangle \,(t)={\rho }_{NV}(t)\otimes {\rho }_{B}$^27^, where 〈*ρ*〉 (*t*) is the system density matrix averaged over all possible stochastic trajectories, *ρ*_*NV*_(*t*) is the NV centre density matrix, where *ρ*_*B*_ describes the nuclear bath, which is thermal. On these timescales, is possible to derive a dynamical equation for the average NV population $n(t)\equiv \frac{1}{2}+{\rm{Tr}}({S}_{z}{\rho }_{NV}(t))$,

2 $$\dot{n}(t)+\alpha (t)n(t)=\frac{1}{2}\alpha (t),$$

where $\alpha (t)=N\,\frac{1}{4}\,({\gamma }_{x}({\rm{\Delta }},t)+{\gamma }_{x}(2\,{\rm{\Omega }}-{\rm{\Delta }},t)+{\gamma }_{z}({\rm{\Omega }},t))$ and Δ = Ω − *ω*_*N*_ is the HHDR detuning, which vanishes in our set-up. For detailed derivation see Supplementary Material. The rates are defined as

3 $${\gamma }_{\beta }(\omega ,t)={\int }_{0}^{t}\,\langle {A}_{\beta }^{i}(\tau ){A}_{\beta }^{i}(0)\rangle \,\cos \,(\omega \tau )\,d\tau .$$

Notice that in a Markov approximation, possible when $t\gg {\tau }_{c}$, the upper limit of the integral may be extended to infinity. In this regime, the rates *γ*_*β*_(*ω*, *t*) correspond to the power spectra of the fluctuations evaluated at different frequencies, in fact *γ*_*β*_(*ω*, *t* → ∞) = 2*S*_*β*_(*ω*), where *S*_*β*_(*ω*) is the spectral density of ${A}_{\beta }^{i}(t)$. From this perspective, the nuclear thermal motion affects the NV centre as en effective bath whose coupling to the electronic spin is *γ*_*β*_(*ω*, *t*). Further, the Markov approximation implies a time-independent rate, *α*_*M*_ ≡ *α*(*t* → ∞), leading to the average population at time *t*

4 $$n(t)=\frac{1}{2}+\frac{1}{2}\,\exp \,(\,-\,{\alpha }_{M}t).$$

As a result, the NV centre looses its initial polarization at rate *α*_*M*_. We remark that this procedure is similar to that presented in^27^. Nonetheless, due to the rapid motion of the water molecules, there is no net polarization transfer to the nuclear bath because this transfer would require flip-flop processes to dominate over flip-flip type interactions. Yet, the protocol presented here is suitable for nuclear sensing, that is, the NV centre loses its initial polarization even if the nuclei do not get polarized. We remark that the depolarization rate *α*_*M*_ depends on the number of spins inside the NV detection volume, *N*, the correlation time, *τ*_*c*_, and the variance $\langle {A}_{\beta }^{i}\mathrm{(0)}{A}_{\beta }^{i}\mathrm{(0)}\rangle$, which are extensive parameters that in turn depend solely on a set of intensive parameters as $\{{\rho }_{W},{z}_{0},{{\mathscr{D}}}_{W}\}$, with *ρ*_*W*_ the proton density in water, and *z*_0_ the vertical distance from the NV to the sample. These latter quantities can be estimated by measurement, which ensures a fit-free model for the population evolution.

### Second Scenario: Solid water

In the case that water remains solid on top of the surface terminated diamond surface, as predicted in^33,34^, the system evolution is again ruled by Hamiltonian in Eq. (1), but with time-independent interactions since all the involved nuclei are now essentially stationary. Moreover, the internuclear coupling, previously neglected due to the rapid motion, now plays a significant role. The nuclear-nuclear interaction, *H*_*N*–*N*_ may be written as

5 $${H}_{N-N}=\frac{1}{2}\,\sum _{i,j}\,{f}_{i,j}({{\bf{r}}}_{i,j})\,({{\bf{I}}}^{i}{{\bf{I}}}^{j}-3\,({{\bf{I}}}^{i}{\hat{{\bf{r}}}}_{i,j})\,({{\bf{I}}}^{j}{\hat{{\bf{r}}}}_{i,j})),$$

where *f*_*i*,*j*_ is the dipole-dipole interaction strength, and **r**_*i*,*j*_ are the position vectors joining two different nuclei. The enormous number of spins within the detection volume makes the derivation of a close analytical solution for *n*(*t*) challenging, and an exact numerical treatment impossible. In order to gain insight into dynamical behaviour of the system, we make use of the Holstein-Primakoff approximation (HPA)^43^, which considers spins as bosons, such that ${S}^{+}\to {a}^{\dagger }$. When using HPA, the resulting system is Gaussian and therefore its evolution can be efficiently computed employing the covariance matrix, ${{\rm{\Gamma }}}_{i,j}=\langle {a}_{i}^{\dagger }{a}_{j}\rangle$^44,45^. More specifically, the HPA maps a polarized spin into a bosonic mode in its ground state. When the evolution is such that only little amounts of correlations are built up among the system and moreover, the Hamiltonian prevents the bosonic mode to get highly populated, then the HPA is expected to give a satisfactory results. In fact, the HPA has been proven to accurately work for baths composed by highly-polarized spins and it is expected to give satisfactory results even for a thermal spin bath^27,46^. The bosonic Hamiltonian after a HPA and a rotating-wave-approximation reads

6 $$H={\rm{\Omega }}{a}_{0}^{\dagger }{a}_{0}+\sum _{i=1}^{N}\,{\omega }_{i}{a}_{i}^{\dagger }{a}_{i}+\sum _{i=1}^{N}\,{g}_{i}{a}_{0}^{\dagger }{a}_{i}+\sum _{i,j}\,{k}_{i,j}{a}_{i}^{\dagger }{a}_{j}+{\rm{H}}.{\rm{c}}.,$$

with the effective nuclear Larmor frequency ${\omega }_{i}={\omega }_{N}-{\gamma }_{N}\frac{1}{2}{A}_{z}^{i}$, the complex coupling constant ${g}_{i}=\frac{1}{4}({A}_{x}^{i}-i{A}_{y}^{i})$, and *k*_*i*,*j*_ the internuclear coupling strength; the NV centre is labelled with *i* = 0. We remark that non-quadratic contributions have been neglected hence, obtaining a quadratic Hamiltonian characterized by flip-flop interactions among different spins. The evolution equation for the covariance matrix may be obtained easily from the Von-Neumann equation, $\dot{\rho }=-\,i\,[H,\rho ]$, where *ρ* is the density matrix of the system. Using that by definition ${{\rm{\Gamma }}}_{i,j}\equiv {\rm{Tr}}\,({a}_{i}^{\dagger }{a}_{j}\rho )$, it is found that

7 $$\dot{{\rm{\Gamma }}}(t)=-\,{\rm{i}}\,[V,{\rm{\Gamma }}(t)],$$

with *V* such that the Hamiltonian can be written as $H={\sum }_{i,j}\,{a}_{i}^{\dagger }{V}_{i,j}{a}_{j}$. The population the NV centre, now expressed in terms of the bosonic operators, is straightforwardly extracted from Γ(*t*) since by definition ${{\rm{\Gamma }}}_{0,0}(t)\equiv {\rm{Tr}}\,({a}_{0}^{\dagger }{a}_{0}\rho )\equiv n(t)$. Note that in general, the diagonal of the covariance matrix Γ_*i*,*i*_ contains the populations of the different bosonic modes while the off-diafonal terms Γ_*i*,*j*≠*i*_ represent the correlations between the different nuclei. Note that differently than other bosonization techniques, we do not rely on collective mode dynamics^47,48^ to reduce the numerical complexity instead we map each spin to an individual boson and hence, the total number of nuclei, *N*, remains the computational limiting factor. For that reason, only particles inside a given interaction volume are considered. Taking a hemisphere of radius 2*z*_0_ around the NV centre, the most relevant interaction is accounted for and we expect a faithful description of the real dynamics. The latter can be easily confirmed taking as a figure of merit the total interaction inside a volume of radius *R*_*M*_, that is, ${\rm{\Lambda }}({R}_{M})\equiv {\sum }_{|{{\bf{r}}}_{i}|\le {R}_{M}}\,|{g}_{i}({{\bf{r}}}_{i})|$. In our system it is verified that Λ(2*z*_0_)/Λ(*R*_*M*_ → ∞) ≈ 80%.

### Coexistence of phases

A more realistic scenario assumes that only a few layers of ice are stabilized on the chemically modified diamond while the remained of the water persists in the liquid phase. In this situation, the NV centre interacts with both solid and liquid water, with the latter diffusing freely above the stationary ice. An appropriate description of the NV centre is obtained when the two previous methods are combined. The solid phase is represented using HPA bosonization and evolves according to Eq. (7), while the liquid water depolarizes the NV centre as predicted in Eq. (2). The cumulative effect is described by

8 $$\dot{{\rm{\Gamma }}}=-\,{\rm{i}}\,[V,{\rm{\Gamma }}]+\alpha (t)\,\{{\rm{\Delta }},{\rm{\Gamma }}\}+\alpha (t){\rm{\Delta }},$$

where Δ is a matrix with elements Δ_*i*,*j*_ = *δ*_*i*,0_*δ*_*j*,0_, indicating that liquid water affects the NV centre directly. A detailed derivation may be found in the Supplementary Material. The depolarization caused on the ice nuclei by the moving water has been neglected since the internuclear interaction strength is several orders of magnitude smaller than the electron-nuclear coupling. Various works have studied the behaviour of liquid water in the presence of ice^49,50^. Two main differences with respect to water in bulk are found: molecular diffusion is slowed down within few nanometres from the ice layer and molecules tend to be ordered in the immediate proximity of ice. The effect of space-dependent diffusion may be computed via molecular dynamics simulations and modifies *α*(*t*) via its dependence on the correlation function. In conjunction with the polarization loss, the molecular ordering may be examined using NV magnetometry.

### NV magnetometry

In the presence of a magnetic field gradient parallel to the NV centre quantization axis, the nuclear Larmor frequency depends on the relative position between the NV and the nuclei, *ω*_*i*_ = *γ*_*H*_**B**(*z*_*i*_), where *z*_*i*_ is the vertical distance of the nuclei from the NV centre Thus, when some layers of ice are formed all the ^1^*H* in a given layer will precess with the same Larmor frequency, *ω*_*i*_ = *γ*_*H*_**B**(*z*_*Layer*_). In this case, the Rabi frequency, Ω, may be tuned to match the specific Larmor frequency of a desired layer. Providing a high magnetic gradient, such that the detuning between the Larmor frequencies in adjacent layers is large compared to the coupling of a specific layer with the NV centre, the NV will interact exclusively with a two-dimensional bath consisting of all the spins forming a given layer. On the other hand, fast moving nuclei will depolarize the NV centre in a broad region of frequencies. Moreover, in our set-up the effect of liquid water may be neglected for timescales that are short as compared to the depolarization rate, $\tau {\alpha }_{M}\ll 1$. As a consequence, scanning different Rabi frequencies with the NV centre, we can individually identify all layers that have remained solid. The theoretically calculated envelope of the resonant peaks may be estimated as

9 $$n({\rm{\Omega }}={\omega }_{i}({z}_{Layer}))=\frac{1}{2}+\frac{1}{2}\,{\cos }^{2}\,(\sqrt{{\rho }_{2D}^{i}}\frac{\beta }{{z}_{Layer}^{2}}\tau ),$$

where *τ* is a fixed interaction time, *β* is a factor arising from the dipolar strength and ${\rho }_{2D}^{i}$ is the surface density of protons in a given layer. Thus, we can estimate both the thickness of the ice and inter-layer distance. Also, changes of superficial density, ${\rho }_{2D}^{i}$, at the solid-liquid interface as predicted in^49,50^ are measurable within our scheme.

## Results and Discussion

The theoretical prediction made for liquid water can be compared with the numerical simulation performed with HPA for the last two cases, namely those of solid water and coexisting solid and liquid water. Recall that within this regime of parameters, a bosonization method for liquid water is proven to give inaccurate results (see Supplementary Material), while an analytical formula for a solid bath does not exist.

**Figure 2:**
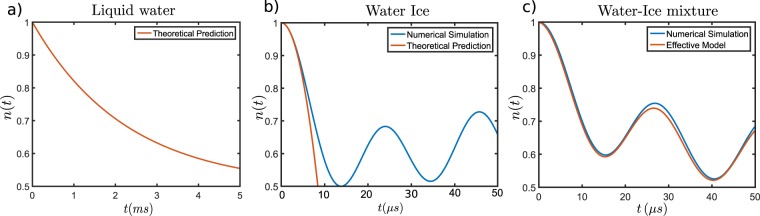
Numerical simulation and theoretical prediction of the evolution of the population, $n(t)\equiv \frac{1}{2}+{\rm{T}}r\,({S}_{z}{\rho }_{NV}(t))$ of a single shallow (*z*_0_ = 3 nm) NV centre interacting with different baths. Note the different timescales. (**a**) Liquid water. The NV centre losses its polarization exponentially at a rate ${\alpha }_{M}^{-1}=2.1\,{\rm{ms}}$, Eq. (4). Notice that for rapid motion, in ms timescale it is verified $t\gg {\tau }_{c}$. The depolarization is a consequence of flip-flop, flip-flip and pure dephasing processes, which are proportional to the depolarization rates cause by the stochastic coupling *γ*_*x*_(Δ, *t*), *γ*_*x*_(2Ω − Δ, *t*), and *γ*_*z*_(Ω, *t*) respectively. (**b**) Water Ice. The NV centre interacts with bulk ice governed by Eq. (7). For short-times an analytical prediction for a spin system is feasible, this gives a good approximation of the interaction time-scale ${\sum }_{i}^{N}\,|{g}_{i}{|}^{2}$ (orange line). The HPA is used to obtain the behaviour at longer time scales (blue line). Coherent oscillations are seen between the NV centre and the protons in the ice. (**c**) Water-Ice mixture. The NV centre interacts with a bath composed by a single ice bilayer and liquid water. The numerical simulation (blue line) was obtained neglecting the interaction with the liquid water. The effective model, (orange line) considers liquid water on top of the ice with space-dependent diffusion coefficient, ${{\mathscr{D}}}_{W/I}(z)$, estimated from^49^. The orange line represents the solution of Eq. (8), where the contribution of moving spins is introduced via *α*(*t*) and the interaction of the static spins appears explicitly in *V*. As can be seen liquid water has a negligible effect during this time-scale due to the fast motion. {For the simulation it has been used: *z*_0_ = 3 nm, *R*_*M*_ = 5.0 nm, ${{\mathscr{D}}}_{W}=2\cdot {10}^{3}\,{{\rm{nm}}}^{2}\,\mu \,{{\rm{s}}}^{-1}$}.

The numerical and theoretical results are both depicted in Fig. 2. On the assumption that water is liquid, a shallow, *z*_0_ = 3 nm, NV centre gets depolarized at a rate ${\alpha }_{M}^{-1}\approx 2.1\,{\rm{ms}}$, where we have assumed that water molecules will exhibit a diffusion constant similar to that of bulk water, ${{\mathscr{D}}}_{W}=2\cdot {10}^{3}\,{{\rm{nm}}}^{2}\,\mu \,{{\rm{s}}}^{-1}$. In fact, at room temperature water molecules diffuse rapidly interacting with the NV during a short time before diffusing away, so their net interaction is weak. In this regime, nuclear polarization is not possible. Indeed, for a net-polarization to happen an imbalance between flip-flop and flip-flip processes between NV and nuclei is required^42^. The effect of flip-flop and flip-flip interactions is here represented by *γ*_*x*_(Δ, *t*) and *γ*_*x*_(2Ω − Δ, *t*) respectively. For fast diffusion, that is, diffusion with a short correlation time, the power spectral density is flat in our frequency range making *γ*_*x*_(Δ, *t*) ≈ *γ*_*x*_(2Ω − Δ, *t*). Hence, the nuclei do not gain polarization. Still, nuclear sensing is possible by measuring the loss in polarization of the NV centre.

On the other hand, when solid water is stabilized on the surface the measurable signal is marked by coherent oscillations in which the NV centre interchanges its initial polarization with the surrounding bath. For short-times the polarization of a spin-system is proportional to $n(t)\approx 1-\frac{1}{2}\,{\sum }_{i}^{N}\,|{g}_{i}{|}^{2}{t}^{2}$, which can be evaluated to coincide with the HPA prediction. In effect, the time scale of the dynamical evolution is determined by ${({\sum }_{i=1}^{N}|{g}_{i}{|}^{2})}^{-\frac{1}{2}}\approx 7.9\,\mu \,s$. In fact, the HHDR condition ensures that $|{\rm{\Omega }}-{\omega }_{i}|\ll 1$, hence the NV centre interacts with each nuclear spin equally. Thus, the short-time dynamics are determined by the collective coupling strength. However, at longer times the different internuclear couplings, *k*_*i*,*j*_ play a major role and estimating a more precise dynamical rate turns intricate. Still, the oscillations persist since for them to be suppressed the spectral density of nuclei in the ice phase must be dense around the Rabi frequency of the NV centre, Ω^51^, which is not true for the current system and thus, the polarization oscillates back and forth. For simulation purpose we have neglect the ice Ih residual entropy^52–54^. In fact, we have considered the protons to be perfectly ordered inside the lattice since entropy effects on the lattice do not significantly affect the qualitative behaviour of signal.

When the two phases coexists, a measurement at HHDR condition will lead again to coherent oscillations between the NV centre and the solid layers. Liquid water depolarizes the NV centre on a longer time-scale. According to^49^, liquid water moves with a space-dependent diffusion coefficient at water-ice interface. It can be approximated as ${{\mathscr{D}}}_{W/I}(z)={{\mathscr{D}}}_{{\rm{\min }}}+({{\mathscr{D}}}_{W}-{{\mathscr{D}}}_{{\rm{\min }}})\,{[1+\exp (-2\kappa (z-z^{\prime} ))]}^{-1}$, where *κ* and *z*′ are some parameters that may be obtained from^49^. This behaviour is easily integrated in our scheme since it only alters the correlation time *τ*_*c*_, which is straightforwardly calculated by numerical means. Computational results in this regime are included in Fig. 2, revealing that even for a space-dependent ${{\mathscr{D}}}_{W/I}(z)$, the depolarization rate is small. Hence, the effects from liquid water are negligible in our timescale. Therefore, a thin layer of ice significantly interacts with the NV centre within few *μ* s.

**Figure 3:**
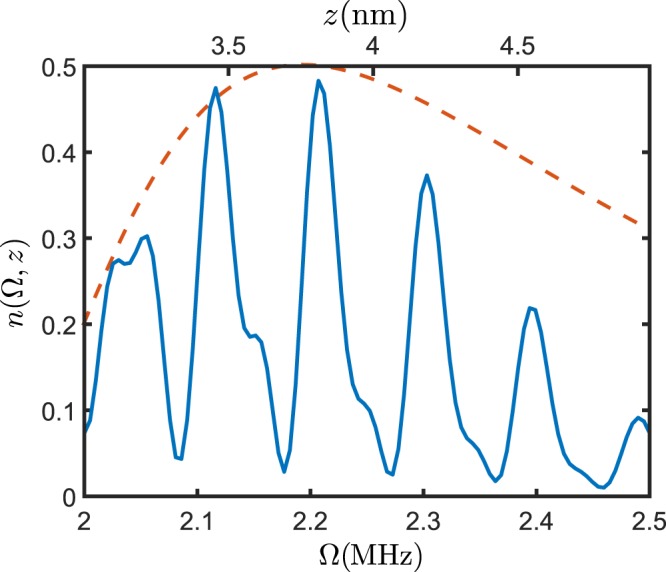
Numerical simulation of NV centre polarization exchange, *n*(Ω, *z*), while interacting with bulk ice in the presence of a gradient field (blue line) compared with theoretical prediction of its envelope Eq. (9) (orange line). Resonant peaks appear when the HHDR condition ${\rm{\Omega }}={\omega }_{i}={\omega }_{N}+\frac{dB}{dz}{z}_{i}$ is fulfilled. Under this condition, the NV centres interacts only with a given layer *i*, which results in a coherent polarization exchange with the bath at a frequency ${\sum }_{j}^{{N}_{i}}\,|{g}_{j}{|}^{2}$, where *j* runs over the *N*_*i*_ spins in *i*-th layer. The distance of each layer to the NV centre is proportional to *ω*_*i*_ and can be easily determined from the measurement. Deviations from the theory for larger distances correspond to an artificial reduction of density due to numerical calculations. When the two phases coexist, only peaks corresponding to solid layers will be seen. {In the model we have assumed the NV depth *z*_0_ = 3 nm, the estimated bidimensional density ${\rho }_{2D}^{i}=15.2\,{\rm{protons}}\,{{\rm{nm}}}^{-2}$ and the spin-locking time *τ* = 30 *μ*s for the continuous driving}.

Finally, in the presence of a strong magnetic-field gradient, we can address individual solid layers. In fact, under a linear magnetic field gradient, *dB*/*dz* = 60 G/nm, which has been proven to be possible in other scenarios^55^, the resolution of the HHDR scheme is sufficient to discriminate different layers as depicted in Fig. 3. Within this scheme structural properties such as the number of layers that remain solid, the interlayer distance or layer density fluctuations can be measured. Importantly, when a layer melts, water particles diffuse away rapidly such that their contribution to the spectra will be only significant at m*s* time-scale, as it occurs with liquid water, thus the resonance peak of a melted layer does not appear in the NV spectra for short interrogation times, conferring high sensitivity to this method. Thus, the number of observed peaks correspond to the number of stabilized layers.

Decoherence processes arising from external sources such as unpaired electron spins at the surface or paramagnetic impurities inside the diamond are neglected in our model. For continuously driven NV centres the limiting time for quantum operations is usually refereed as relaxation time in the rotating frame, *T*_1*ρ*_^29^, which finds its origin in the processes responsible for the dephasing time *T*_2_ in the non-rotating frame^28^. In similar scenarios, for driven shallowly implanted NV centres, this time has been measured to be *T*_1*ρ*_ ≈ ms^15,16^. Based on these considerations and because we are focused on a microsecond time range, we expect *T*_1*ρ*_ relaxation to do not play a relevant role.

## Conclusion

In this work we have demonstrated that detection of different phases of water at room-temperature using shallow NV centres is feasible. More precisely, we have characterized the polarization loss produced on a single NV centre by liquid, solid and liquid-solid large spin baths using HHDR dynamical decoupling. Our theoretical prediction for liquid water has been compared with extensive numerical analysis for solid ice. We have found that on a *μ* s time scale, NV centre polarization undergoes coherent oscillations when interacting with solid water, while interactions with liquid water are negligible in the same time range. Even an ultra-thin ice layer of a few nanometres is detectable using such a protocol. These results permit unequivocal differentiation between solid and liquid water phases. Moreover, using a moderate-strength magnetic-field gradient, the thickness and density of the ice layer can be determined, being able to resolve nanometric structures.

Our approach may find applications in diverse frameworks. This approach can be extended to other solid-liquid phases involving magnetic nuclei, and because our protocol is sensible to density fluctuations, liquid-gas or solid-solid phases are also differentiable. In fact, transitions between different types of water ice, such as Ice Ih and Ice III, are also detectable using NV magnetometry. These transitions are not included in this analysis since they are not expected to occur on a diamond surface. These results open the path towards novel application of NV centres not only as phase sensors but also as valuable tools for nanostructural characterization.

## Electronic supplementary material

Supplementary Information
