# Supplementary material for: Sensing phases of water via nitrogen-vacancy centres in diamond
Source: Sci Rep. 2018 Sep 7;8:13453. doi: 10.1038/s41598-018-31745-3 (PMC6128937; doi:10.1038/s41598-018-31745-3)
Supplement: Supplementary file 1 — Supplementary Information [file 41598_2018_31745_MOESM1_ESM.pdf]

Supplementary Material: Sensing phases of water via nitrogen-vacancy centres in diamond

P. Fernandez-Acebal[1,*] and M.B. Plenio[1]

[1] Institut für Theoretische Physik and Center for Integrated Quantum Science and Technology (IQTS), Albert-Einstein Allee 11, Universitat Ulm, 89069 Ulm, Germany
[*] pelayo.fernandez@uni-ulm.de

Master equation for Liquid Water

In this section we derive the master equation for the liquid water scenario that will lead to Eq.(2) in the main text. Starting from the general Hamiltonian in Eq.(1) of the main text, and under the assumption that for fast-moving particles the internuclear interaction averaged out, we can rewrite the Hamiltonian as

$$H = \Omega S_z + \sum_{i=1}^{N} \gamma_N \mathbf{B}^i_{\text{eff}} \mathbf{I}_i + \sum_{i=1}^{N} g_i(t) S_+ I^i_- + g^*_i(t) S_+ I^i_+ + h_i(t) S_+ I^i_z + \text{H.c.} \tag{1}$$

In the last expression and for the sake of clarity, we have make use of the ladder operators to indicate the possible interactions. Also, we have renamed $g_i(t) \equiv \frac{1}{4}\left(A^i_x(t) + \mathrm{i}A^i_y(t)\right)$ and $h_i(t) \equiv \frac{1}{2}A^i_z(t)$ and $\mathbf{B}^i_{\text{eff}} = \mathbf{B} - \frac{1}{\gamma_H}\frac{1}{2}\mathbf{A}^i\mathbf{I}^i$ is an effective field created on the nuclear spins by the NV centre. First, we consider that we work in high-fields such that $\gamma_N|\mathbf{B}| \gg \mathbf{A}_i \,\forall i$, and thus consider that all the nuclei rotate with identical Larmor frequency ω_N.

Second, in order to treat such a Hamiltonian one may split it into a time independent part, $H_\omega \equiv \Omega S_z + \sum \omega_N I^i_z$, and a time-dependent stochastic part

$$H_{int}(t) = \sum_{i=1}^{N} g_i(t) S_+ I^i_- + g^*_i(t) S_+ I^i_+ + h_i(t) S_+ I^i_z + \text{H.c.} \tag{2}$$

The system will evolve according to the Von-Neumann master equation,

$$\dot{\rho}(t) = -\mathrm{i}\left[H_\omega + H_{int}(t), \rho(t)\right]. \tag{3}$$

This is a stochastic differential equation. Nonetheless, we want to look at the deterministic master equation, that is, averaged over all possible stochastic trajectories. There exist several methods for solving such an equation, in particular we follow[1], which have been used previously in[2,3], obtaining accurate results. For solving the dynamics we go in a interaction picture with respect to H_ω and split the Hamiltonian in a time dependent and time independent part obtaining

$$\tilde{H}_0 = \left\langle \tilde{H}(t) \right\rangle = \sum_{i=1}^{N} \langle g \rangle S_+ I^i_- e^{\mathrm{i}\Delta t} + \langle g \rangle^* S_+ I^i_+ e^{\mathrm{i}(2\Omega+\Delta)t} + \langle h \rangle S_+ I^i_z e^{\mathrm{i}\Omega t} + \text{H.c.}, \tag{4}$$

$$\tilde{H}_1 = \tilde{H}_0 - \tilde{H}(t) = \sum_{i=1}^{N} \mathscr{G}_i(t) S_+ I^i_- e^{\mathrm{i}\Delta t} + \mathscr{G}^*_i(t) S_+ I^i_+ e^{\mathrm{i}(2\Omega+\Delta)t} + \mathscr{H}_i(t) S_+ I^i_z e^{\mathrm{i}\Omega t} + \text{H.c.}, \tag{5}$$

where $\langle\cdot\rangle$ represent the average over all possible stochastic trajectories. We have introduced the detuning $\Delta = \Omega - \omega$ and the hyperfine coupling fluctuations around the average $\mathscr{G}_i(t) \equiv g_i(t) - \langle g \rangle$, $\mathscr{H}_i(t) \equiv g_i(t) - \langle H \rangle$. Also, it is assumed all the particles posses identical average properties, $\langle g_i \rangle = \langle g \rangle \,\forall i$, $\langle h_i \rangle = \langle h \rangle \,\forall i$, which is true for homogeneous motion. We note that in the assumption that $\Delta \approx 0$, and $\Omega \gg \Delta$, we can neglect fast rotating terms form $\tilde{H}_0$ obtaining

$$\tilde{H}_0 \approx \sum_{i=1}^{N} \langle g \rangle S_+ I^i_- + \text{H.c.}, \tag{6}$$

which is a time independent Hamiltonian where all the fast-rotating terms have been neglected. Moreover, in our scenario $\langle g \rangle = 0$.

Always in the interaction picture with respect to H_ω, the master equation may be written as

$$\dot{\tilde{\rho}}(t) = -\mathrm{i}\left[\tilde{H}(t), \tilde{\rho}(t)\right] \equiv \mathscr{L}(t)\tilde{\rho}(t) = \left(\mathscr{L}_0 + \mathscr{L}_1(t)\right)\tilde{\rho}(t). \tag{7}$$

In the last expression we have made use of the Liouville superoperator, $\mathscr{L}_\alpha(t)\cdot \equiv -\mathrm{i}\left[\tilde{H}_\alpha, \cdot\right]$. The presented master equation determines the behaviour of $\tilde{\rho}(t)$ for a specific stochastic trajectory. Yet, we are interested in the average value of the density matrix over all possible stochastic trajectories since it is the solely measurable quantity. Thus, performing a cumulant expansion and following straightforwardly reference[1,3], when $|\tilde{H}_0|\tau_c \ll 1$ and $\left\langle \mathscr{G}^2 \right\rangle \tau_c \ll 1$, which is true for moderate coupling and short τ_c, the average solution of such a system is

$$\dot{\langle\rho\rangle}(t) = -\mathrm{i}\left[\langle H\rangle, \langle\rho\rangle(t)\right] + \sum_{i=1}^{N}\left(\mathscr{H}_i + \mathscr{D}_i^x + \mathscr{D}_i^z\right)\langle\rho\rangle(t), \tag{8}$$

where $\langle\rho\rangle(t)$ is the average of the total density matrix $\rho(t)$ over all possible stochastic trajectories. This is an approximation up to second order in terms of $\langle\mathscr{G}_i^n\rangle\,\tau_c^{n-1}$. Up to this order, the effects of the stochastic motion can be split into three different contributions. A shift to the energy levels

$$\mathscr{H}_i\cdot = \mathrm{i}\Omega_x(\Delta_i, t)\left[S_z - I_z^i, \cdot\right] + \mathrm{i}\frac{1}{2}\Omega_z(\Omega, t)\left[S_z, \cdot\right], \tag{9}$$

with the frequencies defined as

$$\Omega_\beta(\omega, t) = \int_0^t \left\langle A_\beta^i(\tau) A_\beta^i(0)\right\rangle \sin(\omega\tau)\, d\tau. \tag{10}$$

A dissipator originated from flip-flop and flip-flip interactions

$$\mathscr{D}_i^x\cdot = \gamma_x(\Delta_i, t)\left[D\left(S_+ I_-^i\right) + D\left(S_- I_+^i\right)\right]\cdot + \gamma_x(2\Omega + \Delta_i, t)\left[D\left(S_+ I_+^i\right) + D\left(S_- I_-^i\right)\right]\cdot. \tag{11}$$

And the last term which will cause pure dephasing on the NV centre

$$\mathscr{D}_i^z\cdot = \gamma_z(\Omega, t)\left[D\left(S_+ I_z^i\right) + D\left(S_- I_z^i\right)\right]\cdot. \tag{12}$$

We remark that for symmetry reasons, in our set-up it is verified $\langle g\rangle = 0$. That means, there is not a net coherent coupling between NV centre and nuclei, allowing us to express the system state as $\langle\rho\rangle(t) = \rho_{NV}(t)\otimes\rho_B$. The evolution of the NV population $n(t) \equiv \frac{1}{2} + \mathrm{Tr}\left(S_z \rho_{NV}(t)\right)$, may be calculated obtaining

$$\begin{aligned} \dot{n}(t) + \frac{1}{4}N\left(\gamma_x(\Delta, t) + \gamma_x(2\Omega + \Delta, t) + \gamma_z(\Omega, t)\right)n(t) = \\ = \frac{1}{4}N\left(\gamma_x(\Delta, t) - \gamma_x(2\Omega + \Delta, t)\right)n_B + \frac{1}{4}N\left(\gamma_x(2\Omega + \Delta, t) + \frac{1}{2}\gamma_z(\Omega, t)\right). \end{aligned} \tag{13}$$

With n_B the population of a single nuclear spin. When thermal baths are considered, $n_B = 1/2$ and we arrive to a final expression

$$\dot{n}(t) + \alpha(t)n(t) = \frac{1}{2}\alpha(t). \tag{14}$$

With the depolarization rate $\alpha(t) \equiv \frac{1}{4}N\left(\gamma_x(\Delta, t) + \gamma_x(2\Omega + \Delta, t) + \gamma_z(\Omega, t)\right)$.

Equivalence between Master Eq. and dynamical equation for covariance matrix

Now we focus on the derivation of the evolution equation for the covariance matrix that will describe the evolution of the system when a mixture of phases is present. In fact, when the two phases coexist the description of the NV centre dynamics becomes more challenging since we need to combine the evolution given by Eq.(7) in the main text, with the depolarization created by a liquid bath Eq.(2) in the main text. While the former is a equation for bosons, since we have applied the HPA, the latter is a equation for spins. Since we are interested in obtain numerical results, we prefer to translate the equation obtained for moving spins to an equivalent expression for bosons.

We start tracing out the liquid bath in S.Eq.(9) in order to obtain a master equation solely for the NV

$$\dot{\rho}_{NV}(t) = -\mathrm{i}\left[\tilde{\Omega}S_z, \rho_{NV}(t)\right] + \left[\gamma_+ D(S_+) + \gamma_- D(S_-)\right]\rho_{NV}(t). \tag{15}$$

For the sake of simplicity we have introduced the new auxiliary variables, $\tilde{\Omega}$, γ_+, γ_-, we omit its explicit form for a moment since it is not relevant for our porpoise here. Under the influence of the former master equation, the population on the NV evolves as

$$\dot{n}(t) = \gamma_+ - (\gamma_+ + \gamma_-)\, n(t), \tag{16}$$

which correspond to S.Eq.(13), with $n_B = \frac{1}{2}$ and $\gamma_+ = \gamma_- = \frac{1}{2}\alpha(t)$. If now we look at a bosonic system, we have that a master equation of the form

$$\dot{\rho}_{NV}(t) = -\mathrm{i}\left[\tilde{\Omega}\, a_0^\dagger a_0, \rho_{NV}(t)\right] + \left[\gamma_{a^\dagger} D\left(a_0^\dagger\right) + \gamma_a D(a_0)\right]\rho_{NV}(t), \tag{17}$$

leads to a equation for the population as

$$\dot{n}(t) = \gamma_{a^\dagger} + (\gamma_{a^\dagger} - \gamma_a)\, n(t). \tag{18}$$

Therefore, the bosonic system will evolve equivalently to spin system as far as $\gamma_{a^\dagger} = \gamma_+$ and $\gamma_a = 2\gamma_+ + \gamma_-$. For $n_B = \frac{1}{2}$ we can again express these quantities in terms of the depolarization rate, $\gamma_{a^\dagger} = \frac{1}{2}\alpha(t)$, $\gamma_a = \frac{3}{2}\alpha(t)$. Thus, with appropriate rates, we can reproduce on a bosonic system the dynamics generated by a Lindblad dissipator on a spin system.

Provided now that for bosons we write the total Hamiltonian (NV and solid spins) as H, the master equation of the system is

$$\dot{\rho}(t) = -\mathrm{i}\left[H, \rho(t)\right] + \left[\frac{1}{2}\alpha(t) D\left(a_0^\dagger\right) + \frac{3}{2}\alpha(t) D(a_0)\right]\rho(t). \tag{19}$$

This master equation leads to a evolution equation for the covariance matrix of the form of Eq.(8) of the main text. On the other hand, this reflects that a direct application of the HPA on the master equation does not always lead to faithful result. In our case, this forbids us to use the HPA for obtaining results in the case of fast moving nuclei.

NV as magnetometer

In this section we present the derivation of Eq.(9) of the main text. As we are principally interested in the solid part, we use the HPA and treat a system of bosons. Under the action of a high magnetic field gradient parallel to the NV centre quantization axis, the Larmor frequency of a nucleus at a position z_i is just $\omega_i = \gamma_H (B_0 + \lambda z_i)$, where B_0 is a constant magnetic field and λ is the gradient strength $\lambda \equiv dB/dz$. The Hamiltonian for a bosonic system expressed in Eq.(6) of the main text, can be straightforwardly modified resulting in

$$H = \Omega a_0^\dagger a_0 + \sum_{i=1}^{N} \omega_i(z_i) a_i^\dagger a_i + \sum_{i=1}^{N} g_i a_0 a_i^\dagger + \sum_{i,j=1}^{N} k_{i,j} a_i^\dagger a_j + \mathrm{H.c.} \tag{20}$$

For a crystal such as Ice Ih, the ^{1}H nuclei are distributed in layers separated by certain distance d. Therefore, if a given layer seats at a distance $z_L = nd + z_0$ from the NV centre, all the nuclei in this layer will precess with the same Larmor frequency $\omega_i(nd + z_0)$. Moreover, they are detuned from nuclei belonging to adjacent layers, $\tilde{\Delta} = \omega_i(nd + z_0) - \omega_j((n+1)d + z_0) = \gamma_H \lambda d$. If our field gradient is high enough such that $\tilde{\Delta} \gg \sqrt{\sum_{z_i = z_L} |g_i^2|}$, then setting $\Omega = \tilde{\omega}(z_L)$, the effective Hamiltonian may be written as

$$H = \tilde{\omega}(z_L) a_0^\dagger a_0 + \sum_{z_i = z_L} \tilde{\omega}(z_L) a_i^\dagger a_i + \sum_{z_i = z_L} g_i a_0 a_i^\dagger + \sum_{i,j=1}^{N} k_{i,j} a_i^\dagger a_j + \mathrm{H.c.} \tag{21}$$

Note that the sum expands only in a given layer. In the limit of short times, and $g^i > k_{i,j}$, we can neglect the internuclear coupling obtaining

$$H \approx \tilde{\omega}(z_L) a_0^\dagger a_0 + \sum_{z_i=z_L} \tilde{\omega}(z_L) a_i^\dagger a_i + \sum_{z_i=z_L} g_i a_0 a_i^\dagger + \mathrm{H.c.} \tag{22}$$

The latter Hamiltonian may be solved analytically. When at the initial time the NV is polarized while the bosonic bath is in thermal state, we obtain

$$n(\Omega = \tilde{\omega}(z_L), t) = \frac{1}{2} + \frac{1}{2}\cos^2\left(\sqrt{\sum_{z_i=z_L} |g_i|^2} t\right). \tag{23}$$

Recalling now the expression for the coupling strength $g_i = \frac{1}{4}\left(A_x^i + \mathrm{i}A_y^i\right)$, we can compute the sum as

$$\sum_{z_i=z_L} |g_i^2| = \frac{1}{4}\sum_{z_i=z_L} |A_x^i|^2 + |A_y^i|^2 \propto \frac{1}{4}\sum_{z_i=z_L} \frac{\left(x_i^2 + y_i^2\right) z_i^2}{\left(x_i^2 + y_i^2 + z_i^2\right)^5} \approx \frac{1}{4}\rho_{2D}\int_V \frac{\left(x^2 + y^2\right) z_L^2}{\left(x^2 + y^2 + z_L^2\right)^5} dxdy. \tag{24}$$

In the last expression we have introduced the bidimensional density of protons ρ_{2D}. The expression for the population is

$$n(\Omega = \tilde{\omega}(z_L), t) = \frac{1}{2} + \frac{1}{2}\cos^2\left(\sqrt{\rho_{2D}^i}\frac{\beta}{z_L^2} t\right). \tag{25}$$

which corresponds to Eq.(9) of the main text.

References

1. Van Kampen, N. G. *Stochastic processes in physics and chemistry*, vol. 1 (Elsevier, 1992).
2. Bruderer, M., Fernández-Acebal, P., Aurich, R. & Plenio, M. Sensing of single nuclear spins in random thermal motion with proximate nitrogen-vacancy centers. *Phys. Rev. B* **93**, 115116 (2016).
3. Fernandez-Acebal, P. *et al.* Toward hyperpolarization of oil molecules via single nitrogen vacancy centers in diamond. *Nano Lett.* **18**, 1882–1887 (2018).
